# Variation in the Acceptability of Cereal Grasses by *Rhopalosiphum padi* (L.) (Hemiptera: Sternorrhyncha: Aphididae) from Different Geographical Areas in Russia

**DOI:** 10.3390/insects17060593

**Published:** 2026-06-05

**Authors:** Elena S. Gandrabur, Alla B. Vereshchagina, Andrei N. Frolov, Natalia S. Klimenko

**Affiliations:** All-Russian Institute of Plant Protection, Department of Entomology, St. Petersburg-Pushkin, Podbelskogo Highway, 3, 196608 Saint Petersburg, Russia; helenagandrabur@gmail.com (E.S.G.); aphidabver@gmail.com (A.B.V.); klimenko.natalia.01.07@gmail.com (N.S.K.)

**Keywords:** bird cherry-oat aphid, clones, climatic zones, weeds, cultivated cereals, trophic adaptation, reproduction, winging

## Abstract

Global warming and changes in grass biodiversity increase the vulnerability of agricultural crops to the harmful bird cherry-oat aphid, *Rhopalosiphum padi*. This study examined 23 wild grass species, wheat, and maize as hosts for *R. padi.* We studied aphid development on different hosts to estimate reproduction of apterous females and their progeny winging using clones of *R. padi* originated from the Krasnodar Region (Southern Russia) and two districts of the Leningrad Region (Northwestern Russia). Within the framework of “outdoor microcosm” experiments, a highly significant influence of the host, the regional origin of the clone, and the interaction of these factors was detected. The most favorable for *R. padi* development species (along with wheat and maize) were *Poa trivialis*, *Lolium multiflorum*, and *Hordeum jubatum*. The least suitable were *Panicum miliaceum*, *Agrostis capillaris*, *Leymus arenarius*, *Setaria viridis*, *Elymus repens*, and *Bromus erectus*. There was a tendency to decrease aphid reproduction on wild plants with the C_4_ type of photosynthesis. When feeding on less suitable host plants, *R. padi* offspring tend to develop wings quickly, even at low colony density, thereby triggering dispersal. Aphids showed some local trophic specialization: a negative correlation was observed between the offspring of one female during the first 14 days of breeding (P14) and its winging in clones collected from the Leningrad Region, but not from the Krasnodar Region, indicating some spatial heterogeneity in the ecological strategies of aphids. The results obtained may be useful for improving *R. padi* control systems.

## Simple Summary

Global warming is altering the composition of wild grasses, favoring the bird cherry-oat aphid *Rhopalosiphum padi*. This study aimed to assess how different Poaceae species affect aphids’ reproduction and migratory capacity in various Russian regions. For comparison, we utilized *R. padi* clones collected from the Krasnodar and Leningrad Regions, using 23 species of wild grasses, wheat and maize. Aphid development was significantly influenced by plant species, the regional origin of clones, and their interaction. It was revealed that, in addition to cultivated cereals, *Poa trivialis*, *Lolium multiflorum*, and *Hordeum jubatum* are the best hosts for all aphid clones, while *Panicum miliaceum*, *Agrostis capillaris*, *Leymus arenarius*, *Setaria viridis*, *Elymus repens*, and *Bromus erectus* were the worst. It was noted that aphids reproduce poorly on wild plants with C_4_ photosynthesis, such as *Panicum miliaceum* and *Setaria viridis*. On plants less suitable for feeding, offspring of *R. padi* may rapidly become winged, even when the colony is still small, provoking migration activity. The data obtained is of practical importance for the development of plant protection systems against aphids and also allows for more accurate prediction of *R. padi* outbreaks, taking into account the role of wild-growing cereals in aphid diets.

## 1. Introduction

About 25 million years ago, vast grassy areas appeared, including Poales species, and new ecological niches emerged [1,2]. During divergence, grasses successfully adapted to arid conditions. Building on the original C_3_ pathway, they evolved the C_4_ photosynthetic pathway, characterized by high productivity and efficient water use [1,3]. For aphids (Hemiptera: Sternorrhyncha: Aphididae), the speciation of grasses acted as a driver for the increased complexity of their life cycles and trophic interactions [4]. In temperate climates, heteroecy has evolved as a regular alternation between winter (woody) and summer (herbaceous) hosts. Feeding on herbs combined with parthenogenesis (cloning) and polymorphism allows aphids to rapidly increase their numbers, while returning to winter hosts in autumn is necessary for bisexual reproduction [5,6]. In the southern regions, clones of aphids often shift to anholocycly developing year-round only on herbs without bisexual reproduction [7,8,9,10,11,12,13].

The biological features of aphids and their piercing-sucking feeding mode have determined their status as dangerous pests [14]. The damage consists not only of sap depletion but also of the transfer of viral and other infections during migration of winged females from wild-growing grasses to cultivated grain crops [15,16]. Pest control measures are complicated by the tendency of aphids to diversify and generate forms resistant to insecticides and plant varieties [17,18,19].

While the development of aphids on grain crops is well documented, including in Russia, their interactions with numerous species of wild grasses remain poorly understood. This is especially true for weeds, although they are often the dominant components in natural and agricultural lands [9,20,21,22,23,24,25].

In recent decades, climate change has significantly altered natural and anthropogenic landscapes [26], contributing to the spread of wild grasses [27]. Among grasses, in particular, some species of C_4_ plants (*Setaria* spp., *Echinochloa* spp.), as well as some C_3_ plants (*Phleum* spp., *Agrostis* spp.), have expanded their ranges [28,29,30,31,32,33]. It is predicted that global warming will eventually change the structure of plant ecosystems depending on the distribution of C_4_ and C_3_ species [34]. The importance of wild-growing and weedy cereals for ecosystems and the economy began to increase [35,36]. They are used in feed production and breeding of cereals and grasses [37,38,39,40], in studies of trophic relationships of phytophagous and entomophagous insects [41], for phytoremediation [42,43] and landscape design [44,45]. Additionally, they are used in the study of allelopathy [46], serve as raw materials for obtaining extracts with protective properties [47], as biofuel [48] and live mulch [49]. Nevertheless, modern agricultural practices have managed to drastically decrease biodiversity, leaving certain species on the brink of extinction [50,51].

Researchers are increasingly focusing on the role of wild grass biodiversity as reservoirs and overwintering sites for aphids, as alternative hosts during periods of food resource depletion (following harvesting of spring crops, after the use of chemical control treatments) in urban environments and as reservoirs of viral and other infections [24,52,53,54,55,56].

Cereal aphids, as ecologically plastic insects, react quickly to climatic changes and new host complexes. The transformation of the existing trophic relations leads to a change in the phenotypic appearance of populations, including the dynamics of their reproduction and settlement. It is no coincidence that in many countries there is an increase in the harmfulness of aphids in agroecosystems, necessitating improved control of the situation [24,57,58,59,60,61,62,63]. Global economic losses from aphid damage are estimated to be millions of US dollars annually [12,64,65,66,67,68].

The bird cherry-oat aphid *Rhopalosiphum padi* (L.) is one of the most important pests of grain crops, widespread everywhere except in the Arctic zones. This species is a polyphagous insect. In the north of its range, aphids live dioeciously, migrating from winter hosts (*Prunus* spp.) to summer hosts (mainly Poaceae, as well as Cyperaceae, Juncaceae, and Iridaceae). In the south of the range, aphids overwinter in the parthenogenetic phase on grasses [8,9,10,11,12].

Currently, 183 species of Poaceae are recorded as host plants for *R. padi,* and most of these are wild-growing grasses [11]. However, the world literature contains little information on the quantitative assessment of the development of *R. padi* on wild-growing grasses in different geographical latitudes, where the life cycles, host biodiversity, the adaptivity capacity, and virus transmission vary significantly. We hypothesized that the range and suitability of various wild-growing grasses for *R. padi* would vary depending on regional climate characteristics, diversity of local floras, and life cycles in the northwest (across two districts with a clear temperature gradient) and the southern part of the aphid’s range in European Russia.

The ongoing ecological shifts, the increasing harmfulness of aphids and the importance of grasses in agricultural practice have determined the direction of our research. The aim of the work was to study wild grasses as hosts for *R. padi* across geographical areas that differ in climate, floral diversity, and the life cycle of local aphid clones.

## 2. Materials and Methods

### 2.1. Experimental Areas

**Figure 1:**
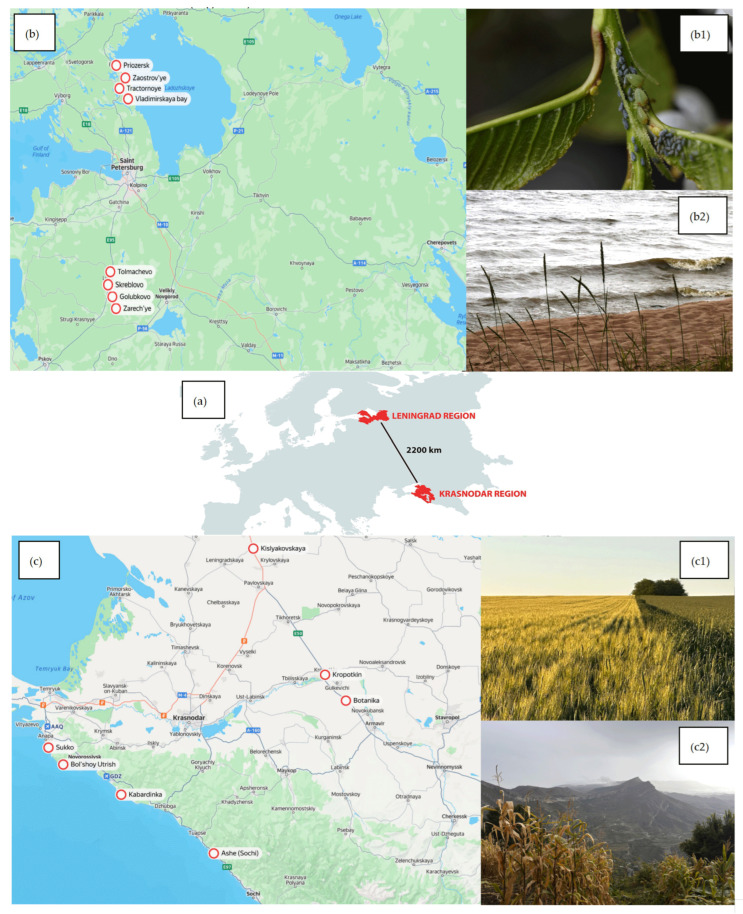
Collection sites of *Rhopalosiphum padi* clones: (**a**) experimental areas. (**b**) Leningrad Region: (**b1**) *Prunus padus* (primary host) with *R. padi*; (**b2**) *Leymus arenarius* (native secondary host for *R. padi*), (**c**) Krasnodar Region: (**c1**,**c2**) grasses (secondary hosts for *R. padi*) in the plains and foothills of the region. Photos by Elena Gandrabur (2024–2025).

The work was carried out in 2025. *R. padi* collections were conducted in two districts of the Leningrad Region (north and south of St. Petersburg) and in the Krasnodar Region (Figure 1). The climatic conditions of the Leningrad Region are considerably colder and more humid than those of the Krasnodar Region, although both regions follow the global warming trend. Cold winters and the widespread distribution of *Prunus padus* L. in the Leningrad Region determine the predominance of *R. padi* with a holocyclic (dioecious) type of life cycle. The area under grain crops and maize as a habitat for aphids in the Leningrad Region is significantly smaller than that in the Krasnodar Region. Aphid clones from northwestern populations (Leningrad Region) were collected from *P. padus* in Priozersk district (the northwestern area) and Luga district (the southernmost area). Thus, the collection sites of *R. padi* belong to different agro-climatic regions of the Leningrad Region [69].

In the Priozersk district (third agro-climatic region of the Leningrad Region), the average daily temperatures in January range from −8 °C to −12 °C, but frosts down to −20 °C to −30 °C occur. The average daily temperature in July ranges from +14 °C to +21 °C. The sum of active temperatures for the period with average daily air temperatures above +10 °C is 1500–1700 °C. Lower temperature sums are observed on the shores of Lake Ladoga and in the lowlands. Luga district (fifth agro-climatic district of the Leningrad Region) is the sunniest in the Leningrad Region. The average daily temperature in January ranges from −9 °C to −4 °C, and in July from +12 °C to +23 °C. The sum of active temperatures for the same period is 1700–1900 °C. The overwintering conditions for plants are the best here. It is no coincidence that there are differences in the taxonomic diversity of weeds, including Poaceae species, between the districts [70]. The annual precipitation in both districts of the Leningrad Region is about the same (600–700 mm) [71].

The Krasnodar Region is characterized by a transition from a temperate continental climate (plains) to a subtropical one (foothill and mountainous zones). Winters are mild here (the average temperature drops to −10 °C, often higher), and summers are hot. The sum of active temperatures for the period with average daily air temperatures above +10 °C in the Krasnodar Region in the plains is 3400–3600 °C, in the foothills 3000–3400 °C [71]. Annual precipitation ranges from 400 to 2500 mm and more. Large areas under winter grain crops and maize are typical of the Krasnodar Region. Under these conditions, aphids mainly live on grasses. Climate warming and changes in agricultural technologies in recent years have caused a rapid increase in the infestation of crops by noxious cereal weeds in the Region [72].

### 2.2. Collection and Maintenance of Aphid Clones

In the Priozersk and Luga districts of the Leningrad Region, aphids were collected on the winter host *Prunus padus*. Samples were taken from colonies of a single clone fundatrices (the overwintering generation) during the migration of emigrants to secondary hosts in early May. In each district, 7 clones of *R. padi* were collected. Of these, in the Priozersk district, clones were collected in Priozersk, Traktornoe, Zaostrovye, and Vladimirskaya Bay. In the Luga district, clones were collected in Zarechye, Golubkovo, Skreblovo, and Tolmachevo. These points are located at a distance of no less than 10–20 km (Figure 1a,b,b1,b2).

A single emigrant was sampled from each clonal colony and transferred into vessels with wheat seedlings under gauze covers on a wooden frame. The spring soft wheat *Triticum aestivum* L. var. *lutescens* cultivar ‘Leningradskaya 6’ was used. Wheat was sown in plastic vessels with a side of 9 cm and a height of 10 cm, 15 grains per vessel.

In the absence of severe winters and the rarity of the primary aphid host *Prunus padus*, anholocycly is characteristic of the Krasnodar population of *R. padi*. All the clones were collected at the end of May. Three clones were collected from the plain part of the region. Of these, one was taken on a maize crop in the Kislyakovskoye village and two on wild-growing *Hordeum murinum* subsp. *leporinum*—near the town of Kropotkin and the village of Botanica. Four clones were collected near the Black Sea coast. Of these, one was picked on wheat in Kabardinka village, and three were gathered on *Hordeum murinum* subsp. *leporinum* located in the villages of Bolshoy Utrish, Sukko and Ashe near Sochi respectively (Figure 1a,c,c1,c2). The minimum distance between aphid collection points was 15 km.

A single apterous female from each location was used to establish clones. Each female was individually placed on seedlings of spring soft wheat (cultivar ‘Leningradskaya 6’) under gauze covers on a wooden frame and transported to St. Petersburg together with her offspring. The wheat was sown as indicated above.

Both the Krasnodar and Leningrad Region clones were maintained until the end of the work by transferring them to vessels with fresh wheat seedlings sown as previously indicated. To do this, approximately 20–25 days after the initial insect infestation, one or two wheat leaves infested with aphids were cut off and placed into new vessels containing the host plants. The transfer of aphids from clones was carried out in a separate room.

### 2.3. Summer Hosts

A total of 25 species of grasses were used as experimental plants (Table 1).

Of these, 9 species are annuals, and 16 are perennial. Four species utilize the C_4_ photosynthetic pathway: *Zea mays*, *Cynodon dactylon*, *Setaria viridis*, and *Panicum miliaceum* [73,74]. Wheat is widely known as a classical C_3_ plant, but shows C_4_ photosynthesis in the developing wheat grain [75]. Wheat and maize were selected as plant species that ensure the successful development of aphids.

Seeds of 16 species of herbs were obtained from the world collection of the N. I. Vavilov All-Russian Institute of Plant Genetic Resources (VIR), the city of Saint Petersburg. The seeds of *Hordeum murinum* subsp. *leporinum* and *Aegilops cylindrica* were collected from plants excavated in the ripening phase in the Krasnodar Region and kept until full maturity in pot culture in St. Petersburg. *Cynodon dactylon* and *Bromus benekenii* were excavated during aphid collection in the Krasnodar Region and were further maintained in St. Petersburg in a pot culture. For experiments, root-sprouting plants in the tillering phase were used. *Leymus arenarius*, *Agrostis capillaris*, and *Poa trivialis* were dug up in the Leningrad Region during the tillering phase before the experiments began. Maize plants were dug up in a field in the Leningrad Region during the phases of vegetative growth, transplanted into vessels and infested with aphids (Table 1).

During the work in the Leningrad Region, route surveys (Figure 1a,b,b1,b2) were conducted to identify wild grass infestations by *R. padi*. Plants infested with aphids were collected. The species identity of the collected grasses, infested by aphids, was determined by botanists—Dr. Luneva N.N. (VIZR), Dr. Mysnik E.N. (VIZR) and Dr. Konechnaya G.Y. (V.L. Komarov Botanical Institute of the Russian Academy of Sciences).

### 2.4. Experiment with Test Plants and R. padi

**Figure 2:**
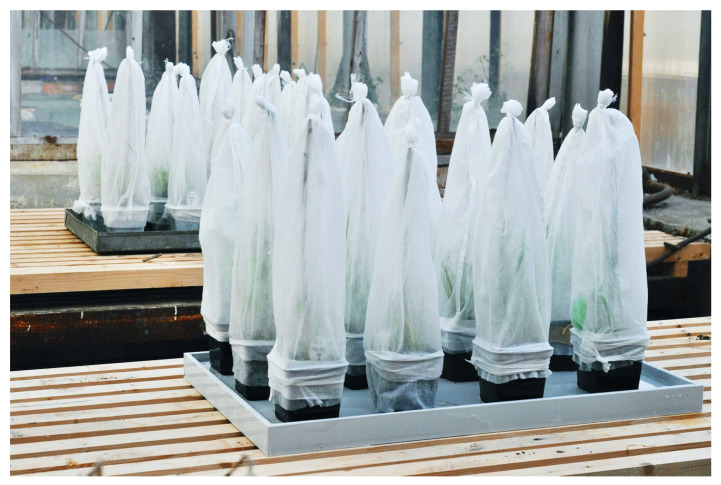
The vessels with gauze cover on a wooden frame. Photo by Elena Gandrabur (2025).

The experiments were conducted on the basis of the All-Russian Institute of Plant Protection (VIZR) in Pushkin (vicinity of St. Petersburg). The experimental insects and plants were kept in an outdoor microcosm (Figure 2), a method that is widely employed in biology [76]. Microcosms were maintained in open-air pavilions with solid roofs. The temperature and humidity conditions were as close as possible to natural ones throughout the experiment. Such factors as entomophages, rain, wind, and food limitation were absent. To prevent pathogen transmission, aphids were transferred from healthy host plants. The health status of the test plants was monitored throughout the study. They remained disease-free. Such a semi-field study allows us to approach real conditions and provides environmental validity, which makes the obtained results more applicable in practice. Air temperature during the experiments was measured using Sper Scientific Digital Min/Max Thermometer (−50 to +70 °C) (Table 2).

Plastic vessels measuring 9 × 9 × 10 cm^3^ were used for sowing plants. Aphid clones were always kept under spunbond insulators on a wooden frame (Figure 2).

(A) Preparation of experimental plants for colonization by aphid clones

To minimize the effect of temperature fluctuations and plant developmental stage on the interpopulation characteristics of aphid clones, plants were sown and populated with aphids in batches of three vessels per plant species. For this purpose, plants of each species were sown in separate vessels with 15–20 seeds each. Vessels similar to those mentioned above were used. The vessels with plant seedlings were covered with insulators even before the aphids were settled. Thus, in each batch, plants of all tested species in the tillering phase were simultaneously populated with one clone from each *R. padi* population. Since seven clones were tested from each district, there were seven experimental batches in total. When sowing each batch of plants, it was taken into account that the tillering phase of grasses occurs 20–30 days after sowing. The sowing of batches of plants was carried out from the first days of May at intervals of 10 days in order to coincide with the summer growing season.

(B) Colonization of test plants by experimental *R. padi* clones

The test plants were always infested by females of the same morphotype (apterous summer female) that had not yet started reproduction. To reduce the transgenerational effect of crowding on offspring composition, experimental females were reared in low-density colonies. For this purpose, 15 females of the respective clone from each population (Krasnodar, Priozersk, Luga) were placed into each of the three vessels with wheat seedlings. The next day, the females were removed. As a rule, after 6–7 days, the born larvae molted into adults, which we used for the infestation of experimental plants. At the same time, wingless females (1–2 individuals) from a single clone of a particular population were alternately placed into each of the three vessels with plants of the same species. A special brush was used to transfer females. The first batch of plants was infested with aphids in mid-June, and the last batch (seventh) in mid-August.

(C) Phenotyping development of *R. padi* on different hosts

For aphid phenotyping, we used estimates of reproduction rate and the ability to disperse as indicators. For each plant species and each clone, there were 7 biological replicates.

The first indicator was based on the counts of the offspring of one apterous female for the first 14 days of reproduction (P14). During this period, some wingless females, not only of the first but also of the second, and rarely of the third, daughter generation, together with the first winged females of the first daughter generation (according to the development pattern of *R. padi*), reach maturity and begin to reproduce. This timeframe allows assessment of the host plant’s impact on the reproduction of the second aphid generation and the development of winged forms—factors that cannot be evaluated with shorter exposure periods on test plants [77].

The second indicator was determined as the ratio of the combined number of alatae females and alatoid larvae (future alatae exules) to the total offspring count, expressed as a percentage.

Counts of the number and determination of the morphotype of the offspring were performed after shaking the insects from the plants onto a sheet of paper. To account for aphids feeding below the soil level, plant vessels were immersed in containers with water so that the upper parts of the leaves were exposed to the air. Aphids feeding below the soil level at the tillering node moved to the top, where they could be easily counted [78]. Aphid counts were carried out in an isolated room to exclude reinfestation.

### 2.5. Statistical Analysis

Variation in P14 and the winging of offspring (developed on different plant species) were analyzed as response variables using mainly R 4.6.0, and also MS Excel 2024, Statistica 13, and PAST 5.3 [79]. Both plant species and region were considered as fixed effects, while clone identity was treated as a random effect. Normality of the data distributions was rejected by the Shapiro–Wilk and Kolmogorov–Smirnov tests, and Levene’s test confirmed the absence of homogeneity of variances. Because heteroskedasticity was detected, a generalized linear mixed-effects model (LME4 package in R 4.6.0) was used to examine variation in both P14 and the winging of aphid offspring. When statistical significance was found, a post hoc Tukey test was applied for multiple comparisons of means, and fixed effects estimates (β) from the linear mixed-effects model were compared. To control for the effect of dependence in correlation calculations, the consistency of aphid clone performance across different grass species (for both P14 and percentage of winging) was estimated using the Intraclass Correlation Coefficient (ICC), calculated with PAST 5.3 (model 2, type: individual). All the original data used in the analysis are available in the attached Supplementary Materials File.

## 3. Results

The characteristics of 25 plant species (Table 1) related to their favorability for reproduction and settlement of aphids of *R. padi* are shown in Table 3 and Table 4.

Variation in P14 was examined using the LME4 package with plant species and region as fixed effects and aphid clone as a random effect. Plant species significantly affected P14 values. As a result, an extremely reliable effect of the host plant, the regional origin of aphids’ experimental clones and the interaction of these two factors on P14 of the offspring of *R. padi* was found (Table 5).

Aphid clone contributed measurable variability (variance = 65.17), although the plant species effect was considerably stronger (Table 5). The highest P14 values were associated with aphid clones with development on *Poa trivialis*, *Triticum aestivum*, *Zea mays*, *Lolium multiflorum*, and *Hordeum jubatum*, whereas *Panicum miliaceum*, *Agrostis capillaris*, *Leymus arenarius*, *Setaria viridis*, *Elymus repens*, and *Bromus erectus* were associated with lower P14 values (Table 6).

Region also significantly influenced P14: between aphid clones originating from the Luga (β = −27.09, P_α_ < 0.001) and Priozersk (β = 17.47, P_α_ = 0.012) districts, both exhibited lower P14 values compared with the Krasnodar Region. However, between clones collected from Priozersk and Luga districts, i.e., within the Leningrad Region, there were no significant differences in P14 values (9.62, P_α_ = 0.53).

Therefore, comparisons among *R. padi* experimental clones from different regions, based on the criterion of colony reproduction (P14) across different hosts, revealed statistically significant groups of plants with varying levels of suitability for clonal reproduction in each region (Table 5 and Table 6). Despite the absence of mortality, the obtained results detected a very significant level of variation among experimental plant species in their favorability for reproduction of *R. padi*, according to indicator P14 (Table 3): the differences between the values of the maximum (259.14) and minimum (13.29) reached almost 20 times the range. The interquartile range (difference between Q_3_ (170.71) and Q_1_ (84.76)), i.e., the 50% range of variation, was 85.9, which is comparable with the median Q_2_ = 108.95. The high level of variation is confirmed by the coefficient of variation estimate, which is equal to 52.4 (calculations were performed using PAST 5.3).

When cultivated maize was excluded, the average reproduction rate of experimental aphid clones reared on wild C_4_ plants (*Setaria viridis*, *Cynodon dactylon*, *Panicum miliaceum*) (mean ± SE = 54.72 ± 13.21) was significantly lower than that on C_3_ plants (123.13 ± 8.48): χ^2^ = 12.81, P**_α_** = 0.025 (Kruskal–Wallis Test).

Variation in offspring winging was also examined using the LME4 package with plant species and region as fixed effects and aphid clone as a random effect. Plant species, region, and their interaction significantly affected offspring winging values (Table 7).

The lowest offspring winging (%) values were associated with aphid clones with progeny feeding on *Poa trivialis*, *Avena fatua*, *Thinopyrum elongatum*, *Thinopyrum intermedium*, *Agrostis capillaris*, and *Phleum pratense*, whereas feeding on *Setaria viridis* and *Panicum miliaceum* was associated with significantly large values (Table 8).

Region of insect collection also significantly influenced offspring winging (%): clones originating from the Luga district (β = 2.14, P_α_ = 0.016) exhibited lower winging values compared with the Krasnodar Region, but not clones from the Priozersk district (β = −0.36, P_α_ = 0.68). There were also no significant differences between clones from Priozersk and Luga districts (β = −1.78, P_α_ = 0.19).

The variation in plant species in the level of wing formation of aphids feeding on them also turned out to be very large-scale: the difference between the maximum (27.5) and minimum (5.5) values (Table 4) was nearly fivefold. The interquartile range, i.e., the difference between Q_3_ (12.12) and Q_1_ (7.78), was 4.34, being almost two times less than the median (Q_2_) equal to 9.00. The high level of variation is confirmed by the coefficient of variation estimate CV = 46.1 (calculated by PAST 5.3).

As for the distribution features of aphid clones according to the P14 criterion and wing formation specificity, they are both characterized by positive skewness. However, the skewness of the P14 distribution (mean ± SE = 0.30 ± 0.46) is much smaller than that of the clones’ distribution according to wing formation (2.02 ± 0.46). As for kurtosis, the differences in the distribution of clones according to the studied traits are more pronounced: for P14, kurtosis is estimated as −0.76 ± 0.90, and for wing formation as 4.53 ± 0.90 (calculated by Statistica 13).

The Pearson correlation between P14 values and the percentage of aphid winging was estimated as a negative and moderately weak value: r = 0.493, P_α_ = 0.012 (calculated by PAST 5.3). This result was obtained based on the use of mean P14 values and the percentage of winging across all clones from all regions and thus represents an overly generalized and simplified characteristic. When a separate regional correlation analysis was performed, taking clonal variability into account, the pattern rather differed. For clones from Krasnodar Region, the correlation was not significant (r = −0.07, P_α_ > 0.05), while for those from the Leningrad Region, it was significant: for Priozersk district, r = −0.270, P_α_ < 0.001; for Luga district, r = −0.375, P_α_ < 0.001.

The degree of similarity among clones in offspring development (P14 criterion) was also estimated using the Intraclass Correlation Coefficient (ICC) (calculated by PAST 5.3, model 2, type: individual). For all clones, the ICC was 0.599 (Confidence Interval (CI): 0.467–0.748). The values by region estimates were similar: for clones from Krasnodar Region, it was 0.607 (95% CI: 0.451–0.764), from Priozersk district—0.733 (95% CI: 0.603–0.849), and from Luga district—0.722 (95% CI: 0.589–0.842). This means that there is a similar ranking of host plants based on their suitability for reproduction among different clones, regardless of region.

However, when it comes to the criterion of winging in offspring, clones show a different pattern of similarity. The ICC for all clones was rather low—0.237 (95% CI: 0.144–0.395). Regional differences were more pronounced, with the highest value for clones from the Priozersk district—0.658 (95% CI: 0.512–0.799), followed by those from the Luga district—0.474 (95% CI: 0.314–0.660), and the lowest level of similarity was shown by clones from Krasnodar Region—0.242 (95% CI: 0.111–0.438).

## 4. Discussion

The choice of experimental plant species is determined by their wide distribution and ecological significance in the natural and anthropogenic landscapes of Northwestern and Southern Russia. In these regions, there are global trends in the transformation of the species and quantitative composition of weeds: widespread invasions and increased contamination of agroecosystems against the background of climate warming, while reducing biodiversity due to economic activity [29,30,31,32,33,50,51].

Poaceae occupy a leading position in terms of species number among weeds both in the Krasnodar Region (33 species) and in the Leningrad Region (30 species) [80,81]. In the Krasnodar Region, there is an increase in the contamination of agroecosystems with grassy weeds, among which *Avena fatua*, *Setaria viridis*, *Bromus secalinus*, *Elymus repens*, and *Cynodon dactylon* are particularly dangerous [72,80]. During weed monitoring in the Krasnodar Region at the end of May, with temperatures exceeding the climatic norm and reaching 30 °C, *R. padi* colonies were found on *Bromus benekenii* and *Hordeum murinum* subsp. *leporinum* in addition to maize.

In the Leningrad Region, *Setaria viridis*, *Poa pratensis*, *Phlum pratense*, *Alopecurus geniculatus*, as well as invasive *Echinochloa crus-galli*, and *Hordeum jubatum* are widespread. During monitoring of weeds in June, aphids were found in large numbers on *Dactylis glomerata*, *Poa trivialis* and *Poa annua.* There is evidence of a richer weed species diversity in the more favorable climate of Luga than in the Priozersk district [82,83,84]. However, during the hot summer of 2025, the highest diversity of wild grasses colonized by *R. padi* was recorded in the Priozersk district, including the moist coastal areas of Lake Ladoga, where the maximum air temperature reached 26 °C. Here, aphids fed on wild-growing plants, specifically *Leymus arenarius*, *Elymus repens*, *Alopecurus geniculatus*, *Alopecurus pratensis*, *Dactylis glomerata*, *Agrostis capillaris*, *Agrostis gigantea*, *Phleum pratense*, *Calamagrostis epigejos* (L.) Roth, *Anthoxanthum odoratum* L., *Lolium perenne*, *Milium effusum* L., *Festuca ovina* L., and *Poa nemoralis* L. In September, significant numbers of aphids were found on maize, *Poa trivialis* and *P. annua* in both regions. Among weeds, some species, such as *Elymus repens*, are widely distributed [85,86]. At the same time, there is a need to monitor the decline in diversity of local weeds, which is primarily associated with a high level of weed control in crops [83,84].

However, Poaceae species are widely distributed in both regions as fodder, hay, pasture, and ornamental crops (Table 1) [84,87]. Despite the dynamic situation with the spread of grasses and the extensive list of known hosts of *R. padi* [11], information about their trophic significance for the pest in the literature remains fragmentary [53,78,88,89,90]. This creates a significant gap in the understanding of the mechanisms behind the formation of these pest reservoirs in natural ecosystems. Therefore, the present study aims to determine the suitability of 25 species of Poaceae as hosts for 21 *R. padi* clones originating from different climatic zones in European Russia. Cereal species used in experiments are widespread and have important ecological significance both in Russia and abroad.

The use of the offspring abundance index for the first 14 days of the reproductive period (P14) proved sufficiently sensitive for the comparative analysis and ranking of wild grasses according to their suitability for the formation of pest foci. All experimental clones were able to reproduce and wing themselves to varying degrees on plants of each species used (Table 3 and Table 4). No mortality was detected among aphids. The absence of mortality proves the wide ecological plasticity of the studied clones and confirms the status of *R. padi* as a polyphagous insect.

**Figure 3:**
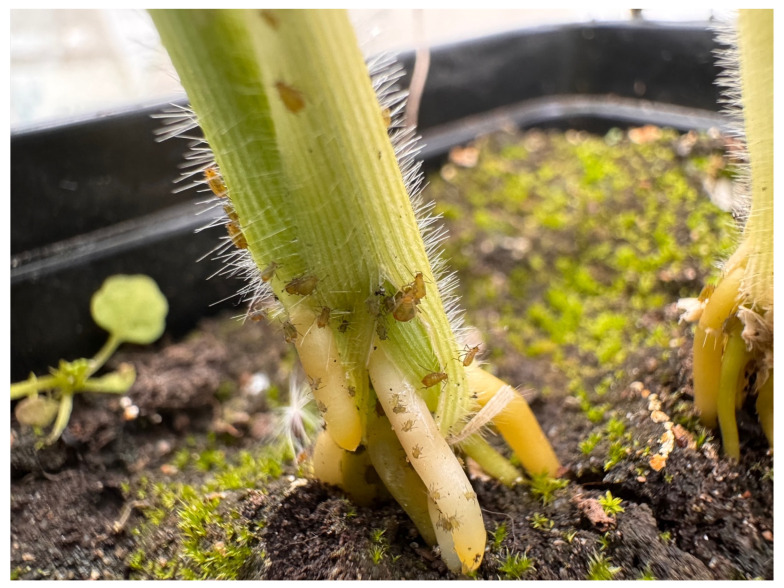
*Rhopalosiphum padi* on aerial roots of *Panicum miliaceum*. Photo by Elena Gandrabur (2025).

The species of wild-growing grasses that are most suitable for breeding *R. padi*–*Poa trivialis*, *Lolium multiflorum*, and *Hordeum jubatum* have been identified (Table 3 and Table 6). These species are most likely to be colonized first. Unfavorable hosts for *R. padi* include *Panicum miliaceum*, *Agrostis capillaris*, *Leymus arenarius*, *Setaria viridis*, *Elymus repens*, and *Bromus erectus.* Among them, aphids multiplied the worst and quickly became winged on *Panicum miliaceum*. Small colonies were formed here only on aerial roots (Figure 3).

It can be assumed that such a topical distribution of aphids is caused by a strong pubescence of plants; however, for example, on *B. secalinus*, which also has pubescence, aphids formed rather large colonies on leaves and stems. Consequently, not only pubescence but also the chemical plant defense of *P. miliaceum* and other hosts were both crucial for the development of aphids. Similar conclusions were previously cited in the literature on host plant resistance [64,91]. Despite their low nutritional value, the least favorable plants are important in the ecology of *R. padi* by functioning as refuge and alternative hosts. Plant species classified in the intermediate group according to their propensity for reproduction of *R. padi* should be kept under observation. Their widespread distribution and important economic importance create favorable conditions for the formation of aphid outbreak centers (Table 1) [82,87,92,93,94,95]. Of these, *Dactylis glomerata* and *Phleum pratense* in Poland [88], *Lolium perenne*, *Dactylis glomerata*, and *Phleum pratense* in Britain [89], and *Lolium perenne* and *Festuca rubra* in the Northwestern United States [96] have already been mentioned as hosts for *R. padi*. Aphids from both climatic zones reproduced by feeding on *Thinopyrum elongatum* (Table 3). This species grows in the European part of Russia and is characterized by high stress resistance to heat and high yields. *T. elongatum* is used as a forage crop in Southern Russia and as an energy raw material in Spain and Canada [48,97]. It also serves as a host plant for aphids with evidence of resistance to *R. padi* in wheat × *Thinopyrum* hybrids [98,99].

Moreover, a *Fusarium* head blight resistance gene (*Fhb7*) has been isolated from *T. elongatum*, further underlining its agronomic importance [100]. Although this resistance is to a fungal pathogen, its discovery underscores the value of *T. elongatum* as a donor of valuable agronomic traits, further justifying the need to study its interactions with pests as part of a comprehensive risk assessment for its agricultural use.

The above ranking of plant species for aphid reproduction confirms and complements the results of our previous work [78,101]. An additional species of wild grasses has been tested. It was shown that the number of offspring (P14) of *R. padi* on the same types of grasses varied depending on the clone and varied by year [78,101]. Similar results were obtained by other authors, for example, when studying species of the genus *Aegilops* as hosts of *R. padi* [90]. Nevertheless, the species of grasses favorable for aphid reproduction in our studies generally coincided [78,101].

The revealed gradation of plant suitability for reproduction of *R. padi* was not shown to be clearly associated with the perennial or annual life history strategy of the plants. There are unfavorable species among annuals (*Setaria viridis*, *Panicum miliaceum*) and among perennials (*Agrostis capillaris*, *Leymus arenarius*, *Elymus repens*, *Bromus erectus*). The mixed composition of the intermediate group of plant species also indicates the absence of pronounced differentiation on this trait, which is consistent with the results of our previous studies [78]. In research by some other authors, *R. padi* consistently showed higher fecundity on annual cereals [102]. Thus, the quality of the feed resources is specific to each plant species and is not clearly determined by their life cycle.

It is assumed that global climate warming will lead to the expansion of wild grasses habitats with a more efficient C_4_ photosynthesis pathway at elevated temperatures. However, the amount of C_3_ grasses will also increase due to increased photosynthetic productivity in a carbon-rich atmosphere [34,74]. Wheat is traditionally classified as a C_3_ plant, despite the presence of C_4_ metabolic elements in the ripening grain [75]. The C_4_ cycle is also characteristic of the previously tested Echinochloa crus-galli [78]. Both our experiments and studies by other authors reveal a tendency for aphids and other phytophages to exhibit a higher trophic affinity for wild C_3_ plants [103,104]. A statistically significant lower reproduction rate of aphid experimental clones on C_4_ plants compared to C_3_ was confirmed only when wild grasses were used, excluding maize. Maize was excluded from the analysis because, despite being a C_4_ plant, domestication and widespread cultivation have affected its suitability for aphids—unlike in wild C_4_ grasses.

Given the highly significant plant species × clone collection region interaction (Table 5 and Table 7), the clone collection regions differ in the sets of plants that are most and least favorable for aphids, both in terms of P14 and offspring winging (%). LME4 testing of a wide range of wild Poaceae revealed local trophic specialization of *R. padi* populations from different climatic zones. The most favorable plants differed between regions: in the Krasnodar Region, *Zea mays* was the leader; in the Luga district, *Zea mays* and *Poa trivialis*; and in the Priozersk district, *Poa trivialis* and *Lolium multiflorum* (Table 3). This change in which species dominate nicely accounts for the significant region × plant species interaction found using the LME4 package.

Special attention should be paid to *Poa trivialis*, which is considered a common species for Eurasia [105], which is ubiquitous in Northwestern Russia and is classified by our testing as a favorable host for *R. padi*. An additional risk factor is the species *Hordeum jubatum*, which is invasive in the Leningrad Region [106], as it serves as an alternative host for *R. padi*. *Leymus arenarius* is widespread in the coastal regions of Northwestern Russia [107] and can become an important reserve plant for the aphid during dry summers. *Hordeum murinum* subsp. *leporinum* is of great importance in the spread of *R. padi* in the Krasnodar Region: the high reproductive rate of the aphid in our experiment is consistent with the pest distribution on this species of barley in this area. *Bromus erectus*, a component of grass-forb communities of the Krasnodar Region, can also be used by *R. padi* for feeding in the Pskov Region [107,108]. Species such as *Agrostis capillaris*, *Setaria viridis*, and *Elymus repens* have such a wide range [53,80,84,85] that even their low nutritional value may be important in maintaining the abundance of *R. padi*.

The statistically significant effect of ‘plant species × region’ interaction indicates that there is differentiation in the response of aphids’ experimental clones to the quality of host plants, depending on the conditions in the clone’s original habitat. This suggests the possibility of local adaptation in populations of *R. padi* depending on characteristics of their region’s habitat. It should be noted that detailed distribution data for the 25 grass species used in this study are not equally available across the Russian regions studied. This prevents a reliable assignment of each species to “co-occurring” (sympatric) or “non-co-occurring” (allopatric) categories for all aphid clones. Therefore, addressing this question is a valuable direction for future research.

The results obtained are consistent with the data of other authors and indicate that wild-growing grasses with various ranges act as an important factor in the adaptogenesis of *R. padi* clones, as well as of other species of phytophages [13,63,109,110,111].

The harmfulness of aphids is associated not only with a high reproduction rate but also with polyphenism. The appearance of winged individuals in colonies ensures rapid dispersal of aphids and will cause the spread of viral and other plant infections [112].

Regarding offspring winging, as with P14, the set of favorable and unfavorable plant species differed markedly between regions. The most consistent unfavorable effect is shown by *Setaria viridis*, which is among the plants that induce elevated winging in all regions. A particularly strong negative effect in the Priozersk district is exerted by *Panicum miliaceum*, where the winging rate significantly exceeds that on other plants. This is in good agreement with the significant region × plant species interaction found using the LME4 package.

According to our results, wing production in aphid colonies (Table 4) feeding on host plants with intermediate suitability (Table 3) exhibited inconsistent variation. This suggests a complex multifactorial influence on offspring morphogenesis, driven by interclonal and interpopulation variability when feeding on the same plant species, and requires further dedicated research.

The degree of similarity among clones in reproductive success (P14), estimated using ICC, was higher than that for wing formation. For all clones, ICC (P14) = 0.599 (95% CI: 0.467–0.748), whereas ICC (winging) = 0.237 (95% CI: 0.144–0.395). This indicates a greater genetic determination of reproductive success, whereas the tendency to produce winged offspring depends more on external factors and genotype-by-environment interactions. Regional differences in ICC for winging (0.242 for clones from the Krasnodar Region and 0.474–0.658 for clones from the Leningrad Region) may be due to different population histories and selection pressure.

In the Leningrad Region, a negative correlation was revealed between P14 and winging (especially in Luga district clones). This is consistent with a trade-off between reproduction and dispersal: on less favorable host plants, where reproductive success is reduced, aphids switch to producing winged dispersers even in the absence of density stress. In contrast, on high-quality hosts (for example, *Poa trivialis*, *Zea mays*), winging remains low even in large colonies—until population density triggers the aphid-specific crowding effect [113]. A similar relationship for aphids between winging, crowding and the quality of host plants has been noted by other authors [114,115]. However, in the Krasnodar Region, where aphids develop year-round on grasses, such a pattern was not found (r = −0.07, P_α_ > 0.05), confirming that this relationship is not universal. Thus, correlations pooled across all regions may mask the spatial heterogeneity of ecological strategies. Further targeted studies are needed to elucidate the details of these relationships.

The data obtained expand our knowledge of factors determining the reproductive potential and dispersal ability of *R. padi* (Table 3, Table 4, Table 5, Table 6, Table 7 and Table 8). Plant species supporting the local reproduction and dispersal of R. padi from adjacent territories into agroecosystems during the summer were identified. Furthermore, the study determined host plants suitable for aphid survival during the period between spring crop harvesting and winter crop emergence, prior to their remigration to primary hosts. Aphid monitoring should be focused not only on economically important crops but also on weed grasses. Moreover, migration from “poor” hosts may begin earlier than from cultivated cereals, which can serve as an early indicator of the risk of virus spread. Regional differences in winging strategies necessitate density threshold values adapted to each region.

## 5. Conclusions

Certain forage, landscape and weedy Poaceae grasses can serve as alternative hosts for *R. padi*. Underestimating the importance may increase infested areas and affect the aphid abundance and viral transmission. To assess the suitability of 25 Poaceae species for *R. padi* development, we evaluated reproduction and wing formation in the progeny of summer apterous females collected from northwestern (Priozersk and Luga districts) and southern (Krasnodar Region) parts of Russia. Statistical analysis revealed that aphid reproduction and winging are significantly influenced by the host plant, clonal geographical origin, and their interaction. Moreover, a higher Intraclass Correlation Coefficient for aphid offspring number (P14) (ICC = 0.599) compared to winging (ICC = 0.237) seems to indicate greater genetic determination of fecundity, whereas the tendency to produce winging offspring depends more on genotype-by-environment. The identified plant groups, ranked by suitability for pest maintenance, allow a new assessment of outbreak risks outside grain crops. For all clones, the most favorable species (along with *Triticum aestivum* and *Zea mays*) include *Poa trivialis*, *Lolium multiflorum*, and *Hordeum jubatum*, while *Panicum miliaceum*, *Agrostis capillaris*, *Leymus arenarius*, *Setaria viridis*, *Elymus repens*, and *Bromus erectus* are the least favorable. Grasses with **C_4_** photosynthesis tended to be less favorable. The differences in the local trophic specialization of clones are shown. A negative P14—winging correlation was found in clones from the Leningrad Region (stronger in Luga district) but not in Krasnodar Region, revealing spatial heterogeneity of ecological strategies. It has been shown that on unsuitable host plants, aphid progeny may quickly develop wings, which facilitates the spread of viral infections. Aphid monitoring should include weeds and even poor hosts. Region-specific density thresholds are needed due to differences in aphid winging strategies. These findings are important for predicting virus spread and optimizing pest control.

## Figures and Tables

**Table 1:** List of Poaceae species studied for suitability for development of *Rhopalosiphum padi* (L.) clones.

Species/Cultivar	Application/Economic Importance	Lifespan	Origin of Plant Accessions ^(^*^)^
*Zea mays* L., cv. Voronezhskiy 158	grain, fodder	annual	nature
*Hordeum murinum* subsp. *leporinum (Link) Arcang.*	weed, fodder	annual	nature
*Thinopyrum elongatum* (Host) D.R.Dewey	breeding of wheat for grain, for hay and for biofuel	perennial	VIR (44486)
*Thinopyrum intermedium* (Host) Barkworth & D.R.Dewey	breeding of wheat for grain, hay, and fodder	perennial	VIR (37639)
*Cynodon dactylon* (L.) Pers	weed, lawn, pasture, for soil phytoremediation	perennial	nature
*Agropyron cristatum* (L.) Gaertin.	fodder, weed	perennial	VIR (K-51104)
*Bromus benekenii* (Lange) Trimen	not used	perennial	nature
*Aegilops cylíndrica* Host.	ruderal weed, pasture	annual	nature
*Avena fatua* L.	malicious segetal weed	annual	VIR (K-380)
*Setaria viridis* (L.)	weed	annual	nature
*Panicum miliaceum* L.	fodder, food	annual	VIR (K-175)
*Leymus arenarius* (L.) Hochst.	decorative, pasture, for hay, secures the sands	perennial	nature
*Alopecurus geniculatus* L.	weed, pasture	perennial	VIR (K-53402)
*Dactylis glomerata* L.	fodder, lawn, decorative, pasture, for soil phytoremediation	perennial	VIR (48100)
*Phleum pratense* L.	fodder, peatland drainage	perennial	VIR (53504)
*Festuca rubra* L.	lawn, for hay, pasture	perennial	VIR (49911)
*Bromus secalinus* L.	segetal weed in winter rye and wheat crops	annual or biennial	VIR (U-153054)
*Bromus erectus* Huds	for hay, pasture	perennial	VIR (54137)
*Hordeum jubatum* L.	decorative, ruderal weed	perennial	VIR (643814)
*Elymus repens* (L.) Gould	malignant segetal-ruderal weed	perennial	VIR (O-152159)
*Lolium multiflorum* Lam.	fodder, lawn	annual	VIR (34955)
*Lolium perenne* L.	fodder, lawn, for soil phytoremediation, as mulch	perennial	VIR (52599)
*Agrostis capillaris* L.	pasture, lawn	perennial	nature
*Poa trivialis* L.	for hay, pasture, lawn, weed	perennial	nature
*Triticum aestivum* L., cv. Leningradskaya 6	grain, fodder	annual	VIR (64900)

Note. ^(^*^)^ Plant material was obtained either from nature (dug up from the ground by ourselves) or received seeds of accessions from N. I. Vavilov All-Russian Institute of Plant Genetic Resources (VIR) (indication of catalog number of the sample obtained is shown in parentheses).

**Table 2:** Characteristics of air temperature (°C) during the research period.

Month	Ten-Day Average			Monthly		
	First	Second	Third	Average	Min.	Max.
June	17.8	16.7	14.8	16.4	6.5	22.0
July	20.3	26.8	27.3	24.8	11.5	31.0
August	22.8	18.3	15.8	19.0	7.0	28.0

**Table 3:** Estimates of the P14 indicator for *Rhopalosiphum padi* clones (mean ± SE) grouped by plant species within all populations combined and separately according to their origin from Krasnodar Region and two districts of Leningrad Region.

Plant Species, Cultivar	Clones from All Populations Combined	Clones Collected from		
		Krasnodar Region	Leningrad Region	
			Priozersk District	Luga District
*Poa trivialis*	259.14 ± 13.06	215.43 ± 23.39	303.57 ± 11.57	258.43 ± 19.44
*Triticum aestivum,* cv. Leningradskaya 6	236.71 ± 16.32	244.00 ± 26.36	252.00 ± 29.16	214.14 ± 31.39
*Zea mays*, cv. Voronezhskiy 158	231.52 ± 18.18	252.43 ± 19.94	167.57 ± 28.08	274.57 ± 32.47
*Lolium multiflorum*	213.71 ± 15.57	167.71 ± 20.76	254.29 ± 27.40	219.14 ± 24.72
*Hordeum jubatum*	197.62 ± 13.16	194.29 ± 29.36	238.71 ± 15.78	159.86 ± 10.02
*Hordeum murinum* subsp. *leporinum*	185.81 ± 14.52	241.57 ± 27.71	169.29 ± 16.56	146.57 ± 15.71
*Bromus secalinus*	170.71 ± 17.51	238.86 ± 30.26	108.14 ± 8.18	165.14 ± 25.51
*Phleum pratense*	169.14 ± 1124	183.00 ± 27.75	168.86 ± 15.75	155.57 ± 13.53
*Alopecurus geniculatus*	155.48 ± 11.83	139.86 ± 15.93	156.00 ± 13.53	170.57 ± 29.73
*Avena fatua*	152.57 ± 16.66	144.43 ± 24.76	169.43 ± 41.42	143.86 ± 19.37
*Dactylis glomerata*	145.05 ± 10.44	138.71 ± 20.93	171.43 ± 16.42	125.00 ± 13.87
*Thinopyrum intermedium*	111.71 ± 11.51	145.43 ± 30.12	99.29 ± 7.36	90.43 ± 8.60
*Lolium perenne*	108.95 ± 8.65	138.86 ± 15.86	91.00 ± 15.17	97.00 ± 6.38
*Festuca rubra*	103.76 ± 11.72	123.29 ± 30.40	85.29 ± 8.61	102.71 ± 15.93
*Agropyron cristatum*	98.62 ± 9.36	134.71 ± 15.51	87.71 ± 9.48	73.43 ± 14.26
*Bromus benekenii*	98.05 ± 12.68	155.14 ± 16.21	95.14 ± 16.94	43.86 ± 4.81
*Aegilops cylindrica*	96.43 ± 14.44	183.71 ± 7.20	59.57 ± 9.45	46.00 ± 4.89
*Cynodon dactylon*	85.95 ± 9.47	130.00 ± 12.97	75.57 ± 9.39	52.29 ± 10.51
*Thinopyrum elongatum*	84.76 ± 7.06	104.57 ± 8.73	81.71 ± 15.77	68.00 ± 7.72
*Bromus erectus*	75.24 ± 8.29	83.86 ± 16.24	82.14 ± 15.85	59.71 ± 10.57
*Elymus repens*	72.95 ± 6.44	68.43 ± 12.20	58.14 ± 7.92	92.29 ± 10.20
*Setaria viridis*	64.90 ± 6.52	88.00 ± 8.30	43.57 ± 4.57	63.14 ± 13.02
*Leymus arenarius*	35.90 ± 4.37	20.43 ± 2.22	54.29 ± 6.33	33.00 ± 6.98
*Agrostis capillaris*	34.71 ± 4.55	22.86 ± 2.82	51.00 ± 9.95	30.29 ± 5.41
*Panicum miliaceum*	13.29 ± 1.66	14.43 ± 4.48	13.57 ± 2.14	11.86 ± 1.58

**Table 4:** Estimates of winged offspring, % (mean ± SE), for *Rhopalosiphum padi* clones grouped by plant species within all populations combined and separately according to their origin from Krasnodar Region and two districts of Leningrad Region.

Plant Species, Cultivar	Clones from All Populations Combined	Clones Collected from		
		Krasnodar Region	Leningrad Region	
			Priozersk District	Luga District
*Panicum miliaceum*	27.49 ± 4.22	17.11 ± 5.04	48.54 ± 5.10	16.80 ± 3.98
*Setaria viridis*	21.23 ± 2.16	18.74 ± 2.28	21.80 ± 4.40	23.16 ± 4.49
*Leymus arenarius*	17.10 ± 1.59	15.43 ± 2.33	12.79 ± 1.60	23.10 ± 2.76
*Aegilops cylindrica*	15.50 ± 1.53	12.04 ± 2.05	14.01 ± 2.59	20.44 ± 2.47
*Bromus secalinus*	13.42 ± 1.45	18.36 ± 2.80	11.73 ± 1.92	10.17 ± 1.83
*Alopecurus geniculatus*	12.64 ± 1.56	17.33 ± 2.89	15.00 ± 1.26	5.60 ± 1.18
*Agropyron cristatum*	11.61 ± 2.83	18.81 ± 6.84	3.79 ± 1.57	12.23 ± 3.43
*Bromus benekenii*	11.19 ± 1.36	9.81 ± 1.60	14.53 ± 2.89	9.23 ± 2.17
*Dactylis glomerata*	10.46 ± 1.51	14.69 ± 2.52	11.33 ± 2.55	5.37 ± 1.59
*Cynodon dactylon*	10.33 ± 1.76	15.23 ± 2.78	6.20 ± 3.19	9.57 ± 2.49
*Hordeum jubatum*	9.86 ± 2.73	21.30 ± 6.02	2.66 ± 0.98	4.90 ± 1.53
*Lolium multiflorum*	9.84 ± 1.61	6.43 ± 2.15	17.13 ± 2.73	5.96 ± 0.67
*Hordeum murinum* subsp. *leporinum*	9.00 ± 1.74	2.69 ± 0.79	17.16 ± 2.78	7.14 ± 1.93
*Bromus erectus*	8.86 ± 1.31	9.09 ± 1.63	4.34 ± 1.17	13.14 ± 2.59
*Zea mays,* cv. Voronezhskiy 158	8.67 ± 1.42	8.39 ± 1.97	12.21 ± 3.18	5.41 ± 1.48
*Elymus repens*	8.62 ± 2.01	15.10 ± 3.81	9.56 ± 3.09	1.21 ± 0.44
*Festuca rubra*	8.43 ± 0.88	8.54 ± 1.50	9.07 ± 1.19	7.69 ± 1.96
*Triticum aestivum,* cv. Leningradskaya 6	8.22 ± 1.66	14.94 ± 3.33	7.80 ± 1.46	1.93 ± 0.57
*Lolium perenne*	8.17 ± 1.17	8.29 ± 1.83	3.33 ± 0.76	12.90 ± 1.44
*Phleum pratense*	7.40 ± 1.59	11.44 ± 4.21	4.03 ± 0.71	6.74 ± 1.51
*Agrostis capillaris*	7.18 ± 1.38	5.33 ± 1.47	6.09 ± 2.57	10.11 ± 2.85
*Thinopyrum intermedium*	6.96 ± 1.49	10.70 ± 3.87	5.89 ± 1.62	4.29 ± 0.98
*Thinopyrum elongatum*	6.86 ± 1.45	2.06 ± 0.68	12.26 ± 2.86	6.26 ± 1.87
*Avena fatua*	6.42 ± 0.89	5.86 ± 1.40	7.61 ± 1.54	5.79 ± 1.82
*Poa trivialis*	5.51 ± 0.85	3.89 ± 1.14	3.69 ± 0.77	8.97 ± 1.55

**Table 5:** Significance of fixed effects (plant species, region of origin of clones, and their interaction) as estimated using the LME4 package for variation in *Rhopalosiphum padi* offspring number during the first 14 days of reproduction (P14).

Source of Variation	df	F	P_α_
Plant species	24	41.96	<0.0001
Clone collection region	2	14.63	<0.0001
Plant species × Clone collection region	48	3.55	<0.0001

**Table 6:** Plant species fixed effects (β) using the LME4 package for offspring number (P14) in *Rhopalosiphum padi*: species with significantly higher or lower offspring number.

Plant Species	β	P_α_
Species associated with significantly higher P14		
*Poa trivialis*	+103.67	<0.001
*Triticum aestivum*	+81.24	<0.001
*Zea mays*	+76.05	<0.001
*Lolium multiflorum*	+58.24	<0.001
*Hordeum jubatum*	+42.14	<0.007
Species associated with significantly lower P14		
*Panicum miliaceum*	−142.19	<0.001
*Agrostis* *capillaris*	−120.76	<0.001
*Leymus arenarius*	−119.57	<0.001
*Setaria viridis*	−90.57	<0.001
*Elymus repens*	−82.52	<0.001
*Bromus erectus*	−80.24	<0.001

**Note.** Positive β means significantly higher P14; negative β means significantly lower P14 compared to the reference (other species).

**Table 7:** Significance of fixed effects (plant species, region of origin of clones, and their interaction) using the LME4 package of variation in *Rhopalosiphum padi* offspring winging (%).

Source of Variation	df	F	P_α_
Plant species	24	11.05	<0.0001
Clone collection region	2	4.85	0.008
Plant species × Clone collection region	48	5.71	<0.0001

**Table 8:** Plant species fixed effects (β) using the LME4 package for offspring winging (%) in *Rhopalosiphum padi*: species with significantly higher or lower winging percentages.

Plant Species	β	P_α_
Species associated with significantly lower winging (%)		
*Poa trivialis*	−7.13	0.005
*Avena fatua*	−6.22	0.015
*Thinopyrum elongatum*	−5.79	0.024
*Thinopyrum intermedium*	−5.69	0.027
*Agrostis* *capillaris*	−5.47	0.033
*Phleum pratense*	−5.24	0.041
Species associated with significantly higher winging (%)		
*Setaria viridis*	+8.59	0.001
*Panicum miliaceum*	+14.84	<0.001

**Note.** Positive β means significantly higher P14; negative β means significantly lower P14 compared to the reference (other species).

## Data Availability

The original contributions presented in this study are included in the article/Supplementary Material. Further inquiries can be directed to the corresponding author.

## Supplementary Materials

The following supporting information can be downloaded at: https://www.mdpi.com/article/10.3390/insects17060593/s1, Table S1: The characteristics of plant species related to their favorability for reproduction and winged offspring in *Rhopalosiphum padi* clones from the Krasnodar Region and two districts of the Leningrad Region; Table S2: Number of offspring (P14) of apterae *Rhopalosiphum padi* from clones when feeding on wild grasses utilize the C4 photosynthetic pathway; Table S3: Number of offspring (P14) of apterae Rhopalosiphum padi from clones when feeding on wild grasses utilize the C3 photosynthetic pathway; Table S4: Collection points for aphids and plant species in Leningrad Region; Table S5: Collection points for aphids and plant species in Krasnodar Region.
